# Influence of Chemical Composition and Water Quenching on the Phase Constitution, Microstructure and Dry-Sliding Wear of Zr-10Nb-xMo (x = 0, 2 and 4 wt.%) Alloys

**DOI:** 10.3390/ma19204417

**Published:** 2026-10-20

**Authors:** Julia Zając, Izabela Matuła, Marcin Nabiałek, Krzysztof Aniołek, Grzegorz Dercz

**Affiliations:** 1 Institute of Materials Engineering, University of Silesia in Katowice, 75 Pułku Piechoty Street 1 A, 41-500 Chorzów, Poland; julia.zajac@us.edu.pl (J.Z.); izabela.matula@us.edu.pl (I.M.); krzysztof.aniolek@us.edu.pl (K.A.); 2 Department of Physics, Czestochowa University of Technology, 19 Armii Krajowej Av., 42-200 Częstochowa, Poland; marcin.nabialek@pcz.pl

**Keywords:** zirconium alloys, biomaterials, heat treatment, microstructure, wear

## Abstract

Zirconium-based alloys are considered promising candidates for biomedical applications due to their favorable mechanical properties and corrosion resistance. In this study, Zr-10Nb-XMo alloys (X = 0; 2; and 4 wt.%) were fabricated using a combination of powder metallurgy and arc melting, followed by water quenching, in order to evaluate the effect of molybdenum addition and heat treatment on their microstructure and tribological behavior. Phase composition was analyzed by X-ray diffraction, while microstructural features were examined using optical and scanning electron microscopy. Tribological properties were assessed under dry sliding conditions using a reciprocating tribometer. XRD analysis revealed the presence of two Zr-Nb solid-solution phases for all investigated compositions, with no additional phases detected within the sensitivity of the method. Rietveld refinement showed that water quenching increased the fraction of the hexagonal phase from about 50 to about 60 wt.% and increased the lattice parameters of both phases, whereas Mo addition mainly decreased the lattice parameter of the cubic phase. Increasing molybdenum content and water quenching were associated with changes in microstructural morphology, including the appearance of finer, needle-like features in some quenched specimens. The lowest mean specific wear rate was recorded for the water-quenched Zr-10Nb-4Mo alloy (6.4 × 10^−4^ mm^3^/(N·m)), compared with 10.5 × 10^−4^ mm^3^/(N·m) for the initial Zr-10Nb alloy. The lower mean wear coincided with higher indentation hardness and changes in microstructural morphology. However, the present results do not establish a direct causal relationship between these characteristics. The results suggest that molybdenum addition combined with controlled heat treatment may provide a promising approach for modifying the microstructure and dry-sliding wear behavior of Zr-Nb-based alloys. Further investigations are required to confirm the reproducibility of the observed trends and evaluate their potential biomedical applications.

## 1. Introduction

The world of science is reflected in market trends and undeniably follows them. As of today, medicine is one of the fastest growing branches of the economy. In the case of medical metals and alloys, there is a continuous increase in the market value. In 2023 it was estimated at USD 16.2 billion and speculated to grow at CAGR of 8.7% in the span of 2024–2030. The main drivers identified by the study were rising incidence of chronic disease and aging populations. Among the types of metallic implants, the dental segment is of particular interest [1,2]. These global trends translate into an increasing demand for metallic biomaterials with precisely tailored structural, mechanical, and biological properties.

Among the wide range of metals used in implantology, particular attention has been devoted to titanium which stands out for its high biocompatibility and excellent mechanical properties [3]. Nevertheless, ongoing efforts to further improve implant performance stimulate interest and boost the search for new solutions and potential alternatives, such as zirconium. The proximity of these elements in the periodic table suggests structural likeness that can translate into similar properties. In addition, zirconium offers a range of different and often better properties worth highlighting, especially in the context of the forming passive oxide layer.

Zirconium and its alloys have attracted increasing interest as potential metallic biomaterials because of their favorable corrosion resistance, mechanical properties and the formation of a protective zirconium oxide layer. The surface oxide may contribute to corrosion resistance and limit metal ion release under suitable environmental conditions. In addition, selected zirconium-based alloys exhibit magnetic properties that may be advantageous for reducing artifacts during magnetic resonance imaging. Nevertheless, their suitability for biomedical applications depends on the specific alloy composition, surface condition and biological environment and requires appropriate experimental verification [4,5,6,7,8,9].

### 1.1. Alloying Additives

For biomaterials, alloying additives must exhibit a range of properties related to their potential impact on the body. First of all, they must be biocompatible, and the resulting compounds from their correlation with other elements must not cause inflammatory reactions. From this perspective, alloying elements such as niobium and molybdenum have attracted considerable attention due to their favorable biological response and beneficial influence on Zr-based alloys. Niobium and molybdenum have been investigated as alloying elements in metallic biomaterials because of their potential to modify phase stability, mechanical properties and corrosion behavior. Their effects depend on alloy composition and processing conditions, while the biological suitability of a specific alloy requires independent evaluation [10,11,12,13]. Additionally, molybdenum impacts the grain refinement and increases the yield strength, while niobium stabilizes β-Zr phase and allows the relative ductility of the material to be maintained [12,13]. In addition to chemical composition, the final properties of zirconium-based alloys are strongly impacted by the applied heat treatment, which additionally exerts a significant influence on the correlation between the base element and additions.

### 1.2. Heat Treatment

Heat treatment is one of the most commonly applied technological processes with regard to metals and their alloys. It causes changes in the mechanical and structural properties of materials without influencing their chemical composition and integrity, as the processes take place in the solid form. One form of heat treatment is quenching, which is used to increase the hardness and strength of the alloys by rapidly cooling them from high temperature, characteristic of a material, in a medium like water, oil, molten salts, polymers, other fluids or air. It also allows us to keep metastable structures and phases in the material by preventing them from degrading in an otherwise slow cooling process. In certain cases extremely rapid cooling can result in amorphous metal [14].

Heat treatment and rapid cooling can significantly influence the phase constitution and microstructure of zirconium-based alloys. Depending on alloy composition and the temperature from which quenching is performed, rapid cooling may retain metastable phases or induce diffusionless phase transformations. However, the relevant phase boundaries depend on the concentration of alloying elements. Therefore, the phase constitution at the quenching temperature and the subsequent transformation pathway must be considered individually for each alloy composition. The treatment is often followed by ageing to increase the corrosion resistance of the material. Binary zirconium alloys (e.g., Zr-2.5Nb) subjected to such heat treatment may show reduced creep resistance. Solely quenching of these alloys results in precipitation hardening due to the formation of second-phase particles [15]. Heat treatment of multi-component alloys (i.e., Zircaloy-4) results in the appearance of precipitates consisting of Fe and Cr dissolved in β-Zr, whose size and distribution depend directly on the cooling rate [16,17].

## 2. Materials and Methods

The studied materials were manufactured from commercially available Atlantic Equipment Engineers (AEE) metal powders of Zr (ZR-105, purity > 99%, particle size −20/+60 mesh), Nb (CB-101, purity 99.8%, particle size 1–20 µm) and Mo (MO-101, purity 99.98%; particle size 1–2 µm). Samples identification correlates with chemical composition, as well as the process of heat treatment applied (water quenching—WQ), and were as follows (Table 1):

Based on the selected chemical compositions, two sets of samples were prepared in order to evaluate the influence of heat treatment on the microstructure and functional properties of the alloys.

The elemental powders were initially blended in a Fritsch Pulverisette 7 premium line planetary ball mill under a protective argon atmosphere (Ar 5.0). This step was intended to improve the distribution of the constituent powders before consolidation and subsequent vacuum arc melting, rather than to induce mechanical alloying. Milling was performed at a rotational speed of 100 rpm for 72 h, with a powder-to-ball mass ratio of 1:5. The milling jar and balls were made of hardened stainless steel. Consequently, the possibility of Fe and Cr contamination from the milling media cannot be excluded, as these elements were not specifically quantified in the present study. Nevertheless, our previous investigations conducted under comparable milling conditions indicated that contamination originating from the milling media remained at the parts-per-million (ppm) level. The blended powders were subsequently cold-isostatically pressed at 450 MPa to obtain compact charges suitable for arc melting. Following powder preparation, the consolidated material was subjected to melting in a vacuum arc furnace (VAM) in an Ar-protective atmosphere at a 1.2 bar pressure. Prior to the melting process, a high-purity Ti-getter was used to remove residual gases from the chamber. In order to ensure proper homogenisation of the alloy, the obtained buttons were flipped over and remelted five times for 60 s per melting cycle. Following arc melting, the specimens were enclosed in quartz ampoules to prevent oxidation during subsequent heat treatment. The specimens were divided into two groups to evaluate the influence of cooling conditions on their phase constitution, microstructure, and tribological properties. Both groups were heated at a rate of 10 °C/min and annealed at 900 °C for 12 h. The first group was cooled under [specify actual cooling conditions], whereas the second group was rapidly quenched in ice water following the breaking of the quartz ampoules. This approach enabled a direct comparison of the effects of different cooling conditions on the investigated alloys.

For the analysis, specimens were prepared using a standard metallographic procedure: grinding with abrasive papers and polishing by rotary discs covered with felt, moistened with Struer’s colloidal OP-S silica suspension with a grain size of 0.04 µm. Etching was performed in a solution consisting of 45 mL 90% lactic acid, 45 mL 65% nitric acid (V) and 8 mL 40% hydrofluoric acid.

The microstructure of samples was observed using an optical microscope (OM) Olympus GX51 (Tokyo, Japan) under ×500 magnification and a scanning electron microscope (SEM) JEOL JSM-6480 under ×1000 magnification. Microstructural observations were performed to correlate phase composition with morphological features of the alloys.

The qualitative X-ray diffraction (XRD) analysis was carried out with a Phillips X’Pert Pro diffractometer (Almelo, The Netherlands) using an X-ray tube with copper anode (λ CuKα = 1.54178 Ǻ) with the following operation conditions: 30 mA and 40 kV. Registration was performed with the step-scanning method in steps of 0.04° in a range of 10–140° (2θ). The quantitative analysis was performed by Rietveld refinement from which the phase fractions and lattice parameters were determined. The results were analyzed using X’Pert High Score Plus software (v. 3.0d) and the ICDD database-PDF5+.

Instrumented indentation hardness, expressed as Vickers hardness (HV_IT_), was measured using a Micro Combi Tester MCT3 (Anton Paar, Switzerland) in accordance with the relevant standard (ISO 14577) [18]. A Vickers indenter was used with a maximum applied load of 500 mN, a data acquisition frequency of 10 Hz, and a holding time of 10 s at maximum load. Ten measurements (*n* = 10) were performed for each sample.

The tribological behavior of the alloys was investigated under dry sliding conditions using an Anton Paar TRN Tribometer in linear reciprocating motion. The tests were performed under a normal load of 10 N at a frequency of 2 Hz, with a stroke length of 10 mm, a maximum linear speed of 6.28 cm/s, and a total sliding distance of 100 m. The temperature and relative humidity were 21 ± 1 °C and 40 ± 5%, respectively. ZrO_2_ was used as the counter-body material. The mean cross-sectional area of the wear tracks (A_w_) was determined using a Mitutoyo Surftest SJ-500 contact profilometer. One tribological test was performed for each material condition (*n* = 1). Eight profilometric cross-sections were measured at different locations along the wear track to characterize the local variability of material removal within the same specimen. The standard deviations reported for the wear-track area and specific wear rate therefore represent within-track measurement variability and not the reproducibility of independent tribological tests.

## 3. Results

### 3.1. Analysis of Phase Composition by X-Ray Diffraction

**Figure 1:**
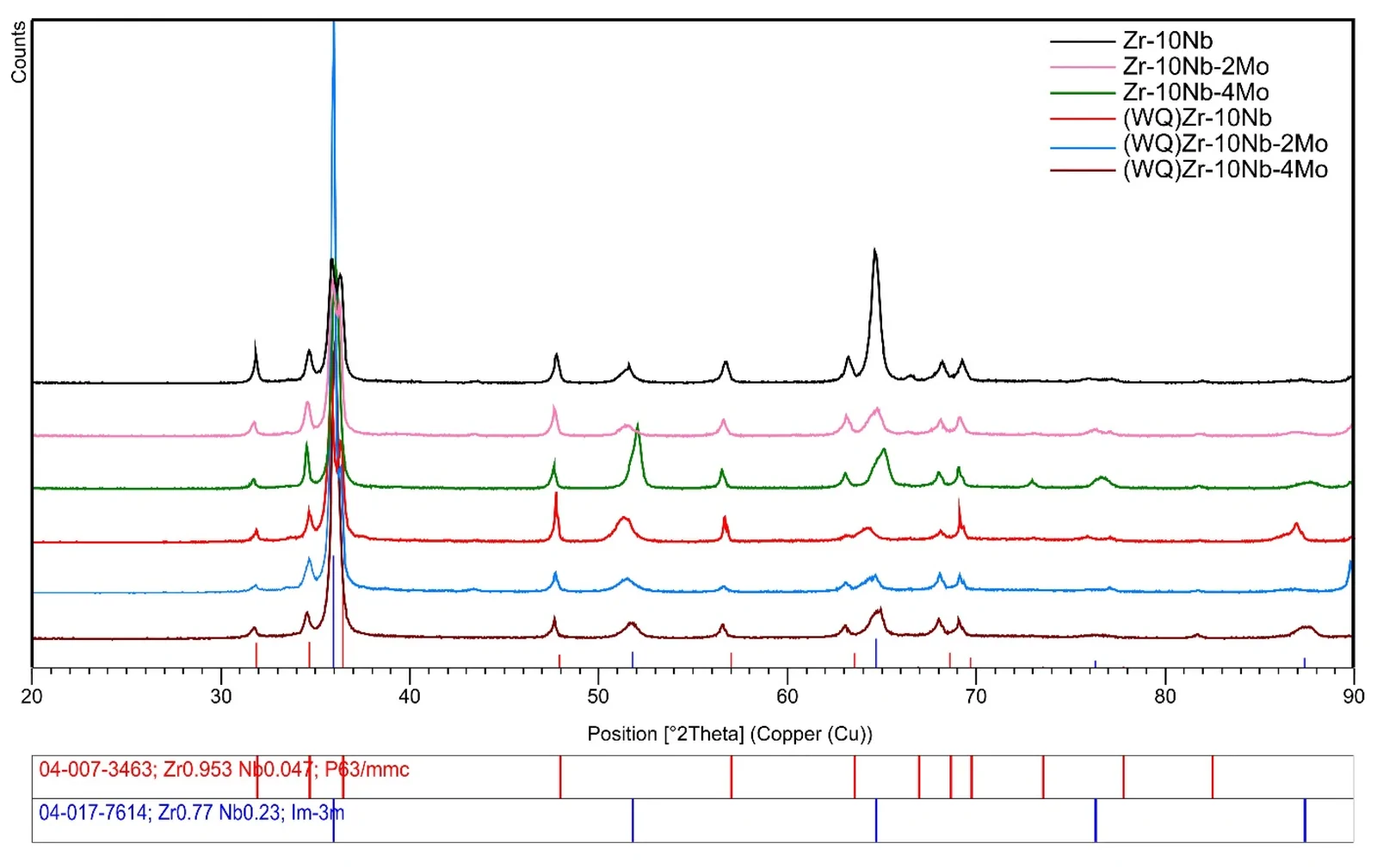
X-ray diffraction patterns of the identified phases of the studied samples.

The XRD study of the prepared alloys constitutes a crucial step in advancing their initial characterization, providing essential insights into their structural properties. The X-ray diffraction patterns of samples resulting from analysis of the phase compositions are presented in Figure 1. Independent of composition, the diffraction patterns reveal the presence of two zirconium-based solid solutions with cubic and hexagonal crystal structures. The diffraction patterns were indexed using two reference structures corresponding to a body-centered cubic (bcc) Zr-Nb solid solution (ICDD PDF 04-017-7614) and a hexagonal close-packed (hcp) Zr-Nb solid solution (ICDD PDF 04-007-3463). The nominal compositions associated with these reference patterns should not be interpreted as experimentally determined compositions of the phases present in the investigated alloys. The actual partitioning of Nb and Mo between the identified phases was not determined in the present study. Their contribution varied among certain samples which is associated with the changing chemical composition as well as performed water quenching. The quantitative phase analysis performed by the Rietveld method is summarized in Table 2 and Table 3. In the initial samples, the cubic and hexagonal phases are present in approximately equal amounts (about 50 wt.%), irrespective of Mo content. After water quenching, the fraction of the hexagonal phase increased to about 60–62 wt.%, while that of the cubic phase decreased to about 38–40 wt.%. The lattice parameters of both phases also increased after quenching (Table 2), which is consistent with a change in their chemical composition and the presence of internal stresses. Since α and α′ have the same space group (P6_3_/mmc), the hexagonal phase in the quenched samples cannot be unambiguously identified as equilibrium α or α′ martensite by XRD alone. It cannot be excluded that traces of Mo-rich phases are present in the Mo-containing samples, but if so, they are at the detection limit of XRD and were not considered further.

Increasing molybdenum content was associated with changes in the relative intensities of selected diffraction peaks assigned to the hcp Zr-Nb (Zr_0.953_Nb_0.047_ P6_3_/mmc) solid solution, particularly when compared with the Mo-free alloy. However, the Rietveld analysis showed that the phase fractions in the initial samples remained practically unchanged (about 50 wt.% of each phase, Table 3), so the observed differences in peak intensities are not related to a change in phase content and may result from different crystallite orientation and/or lattice distortion. In contrast, samples subjected to quenching show a decline in peak intensity for both identified phases. Quenching may cause the retention of certain phases in the solution [14]. In the quenched samples, the Rietveld analysis indicated a redistribution of the phase fractions in favor of the hexagonal phase (about 60–62 wt.% vs. about 50 wt.% in the initial samples) and an increase in the lattice parameters of both phases (Table 2 and Table 3). The intensity of the peaks depends not only on the orientation and crystallographic arrangement of atoms, but also on the internal strain. As rapid cooling during quenching is a strain-inducing process, lattice parameters are affected, leading to peak shifts and broadening [19]. Therefore, the lattice parameter changes may be related to the quenching-induced strain and/or to changes in the chemical composition of the phases. These contributions were not separated in the present study.

### 3.2. Microstructure Observations

**Figure 2:**
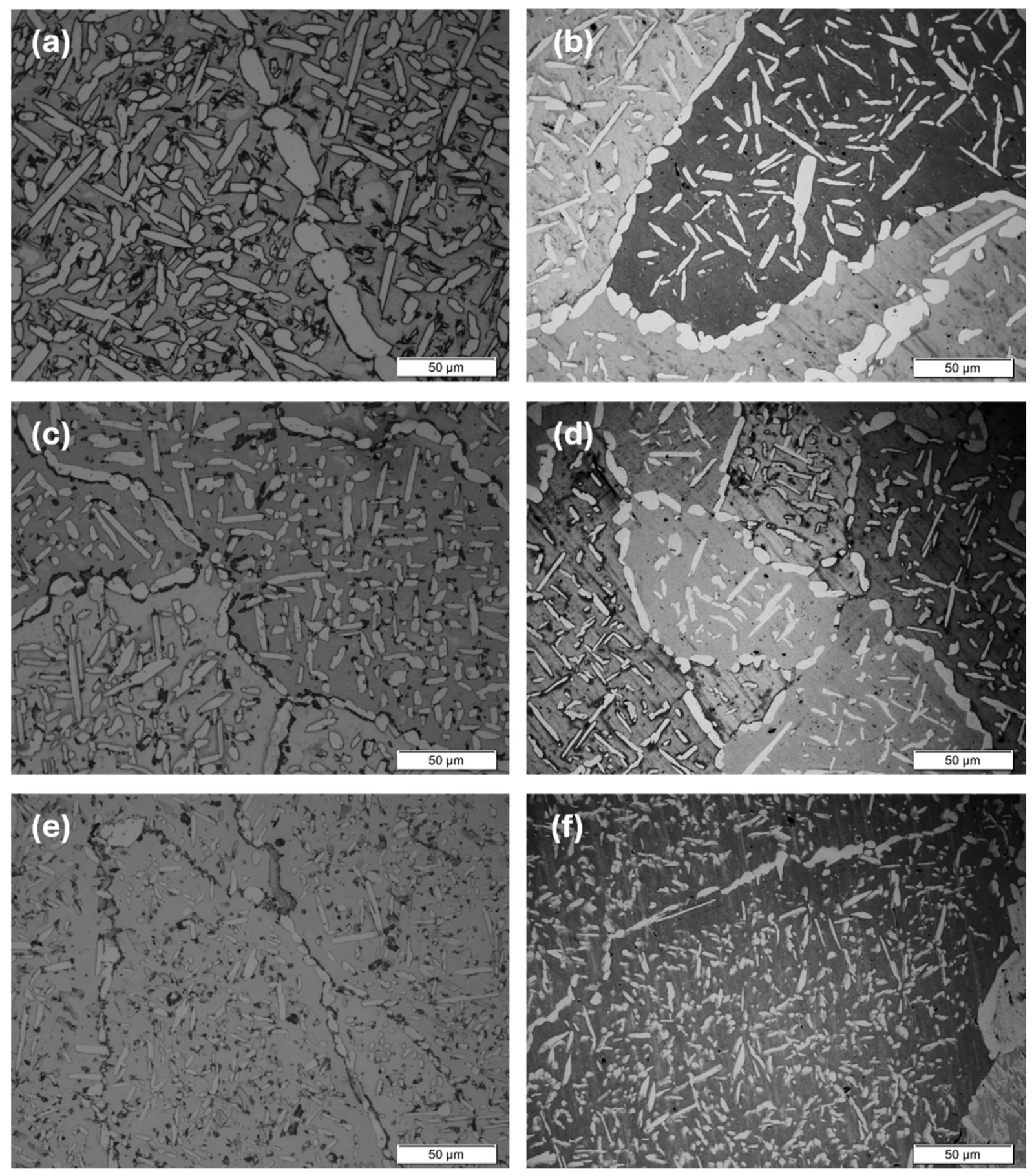
OM images (×500) of samples: (**a**) Zr-10Nb, (**b**) (WQ)Zr-10Nb, (**c**) Zr-10Nb-2Mo, (**d**) (WQ)Zr-10Nb-2Mo, (**e**) Zr-10Nb-4Mo, (**f**) (WQ)Zr-10Nb-4Mo.

**Figure 3:**
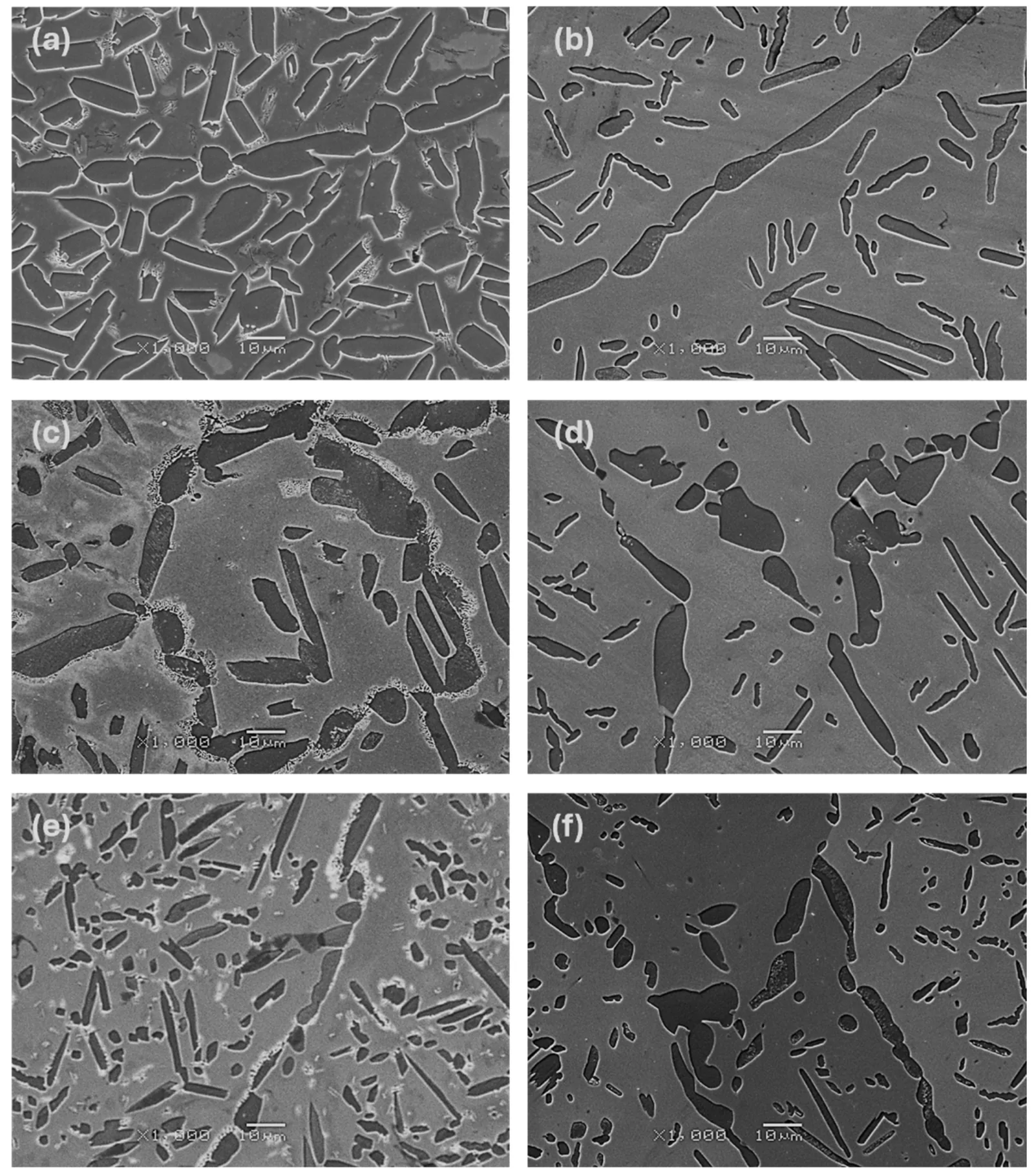
SEM images (×1000) of samples: (**a**) Zr-10Nb, (**b**) (WQ)Zr-10Nb, (**c**) Zr-10Nb-2Mo, (**d**) (WQ)Zr-10Nb-2Mo, (**e**) Zr-10Nb-4Mo, (**f**) (WQ)Zr-10Nb-4Mo.

Microstructural observations using optical microscopy (OM) and scanning electron microscopy (SEM) were performed to complement the phase analysis and to elucidate the effect of molybdenum addition and quenching on the morphology and distribution of structural constituents (Figure 2 and Figure 3).

In the as-annealed condition, all investigated alloys exhibit a heterogeneous microstructure composed of grains with varying morphology and orientation. Elongated, lamellar as well as near-equiaxed grains are simultaneously present, indicating non-uniform grain growth during solidification and subsequent thermal treatment (Figure 2a,c,e). Such morphological diversity suggests a limited grain boundary mobility and a locally varying driving force for grain growth.

Qualitative examination of the OM and SEM micrographs suggests changes in microstructural morphology with increasing molybdenum content and following water quenching (Figure 2 and Figure 3). In particular, finer elongated and lamellar features can be observed in some Mo-containing specimens. Solute drag may contribute to the suppression of grain-boundary migration, as discussed in the literature for related Zr-based alloys [20]. However, neither grain-boundary segregation nor grain size was quantitatively evaluated in the present study. Consequently, the proposed refinement mechanism should be regarded as a possible explanation rather than an experimentally confirmed process.

In addition to changes in microstructural morphology, contrast variations were observed near some microstructural boundaries in the initial specimens. The intensity of these features appeared to decrease with increasing molybdenum content and following water quenching, particularly in the (WQ)Zr-10Nb-4Mo alloy (Figure 2f). However, their origin cannot be unambiguously determined from the present OM and SEM observations, as no localized chemical or crystallographic analysis was performed to identify the corresponding microstructural constituents.

SEM observations reveal changes in the morphology of the investigated alloys following water quenching (Figure 3). Compared with the initial condition, the quenched specimens, particularly (WQ)Zr-10Nb-2Mo and (WQ)Zr-10Nb-4Mo, exhibit finer, needle-like microstructural features in selected regions. Such morphology may be compatible with products of diffusionless transformations reported in zirconium-based alloys [21]. However, the present OM, SEM and XRD results do not allow the transformation mechanism to be identified unambiguously. Therefore, the observed features are described in terms of their morphology without assigning them to a specific martensitic phase. The increased homogeneity suggests that quenching effectively suppresses phase segregation and promotes a uniform transformation throughout the material volume.

Furthermore, SEM images of quenched samples reveal the presence of fine pores distributed within the microstructure. Their occurrence is likely related to internal stresses generated during rapid cooling, which may induce localized strain accumulation and microvoid formation [22].

### 3.3. Tribological and Mechanical Studies

The instrumented Vickers hardness (HV_IT_) of alloys was investigated to assess the influence of the increasing Mo and water quenching on the mechanical response. Furthermore, the results provide data to establish the microstructure–hardness–wear relationship. The mean results are presented in Table 4.

**Figure 4:**
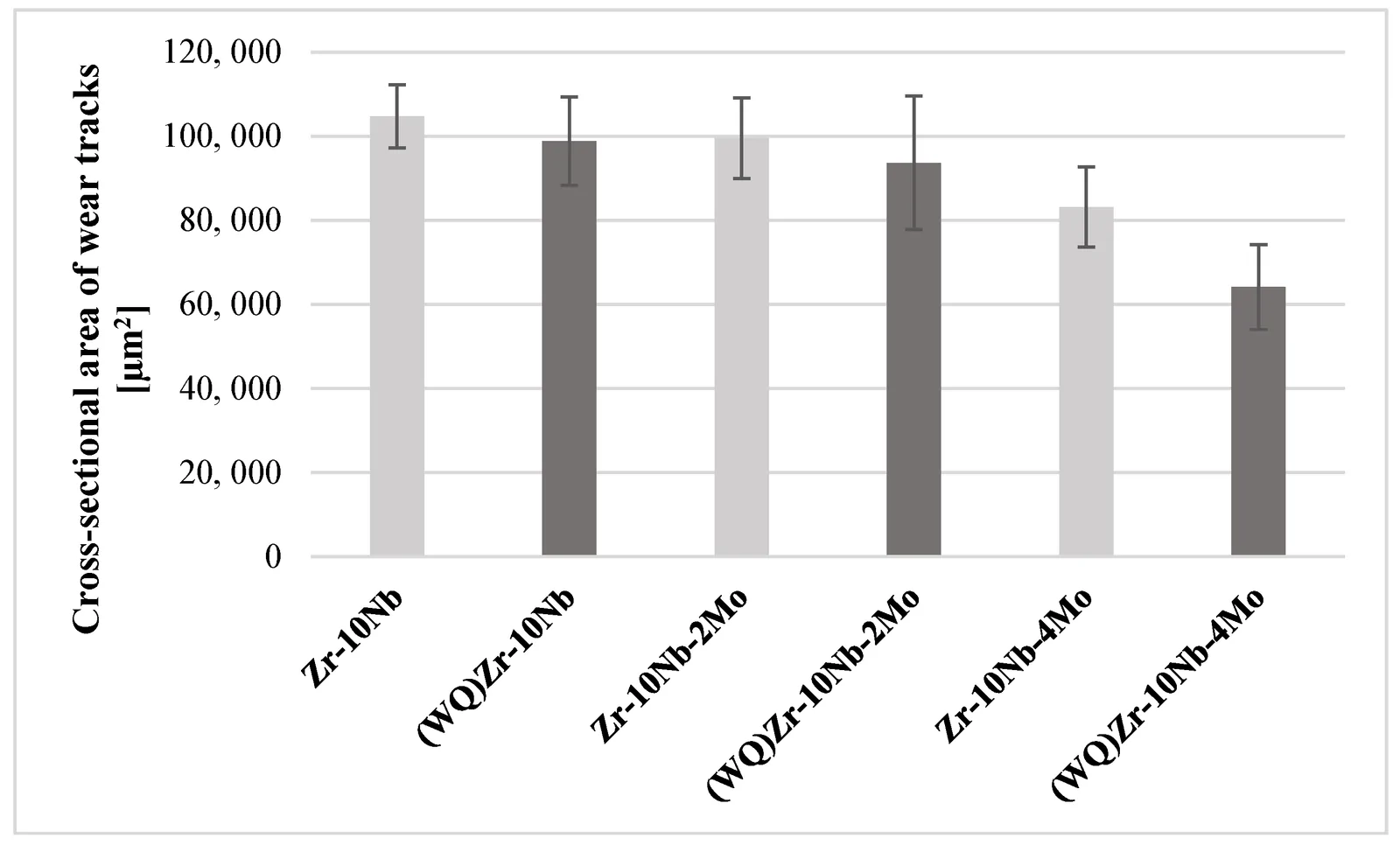
Cross-sectional areas of wear tracks (A_w_) of the tested samples.

**Figure 5:**
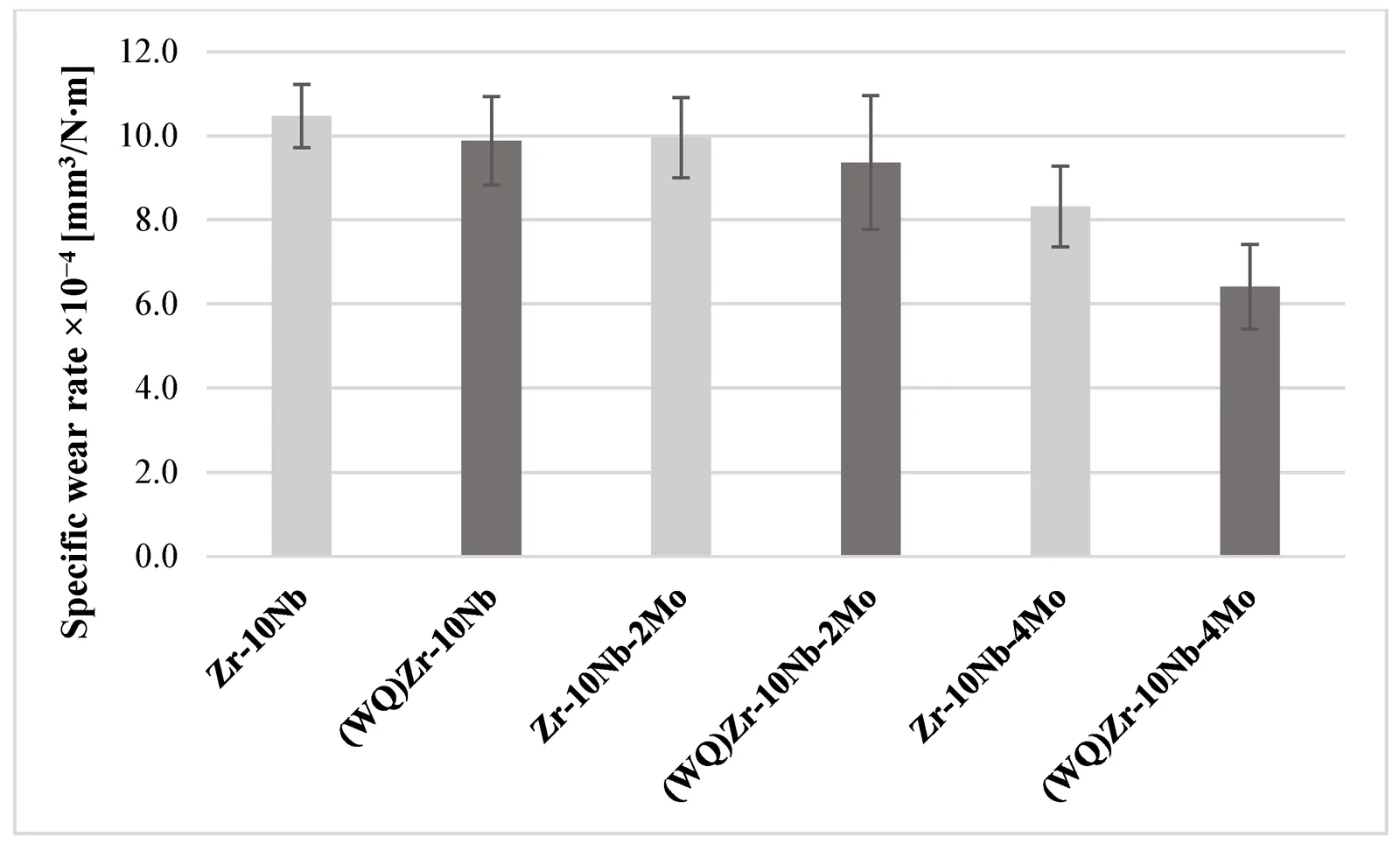
Specific wear rates (w_s_) of the tested samples.

**Figure 6:**
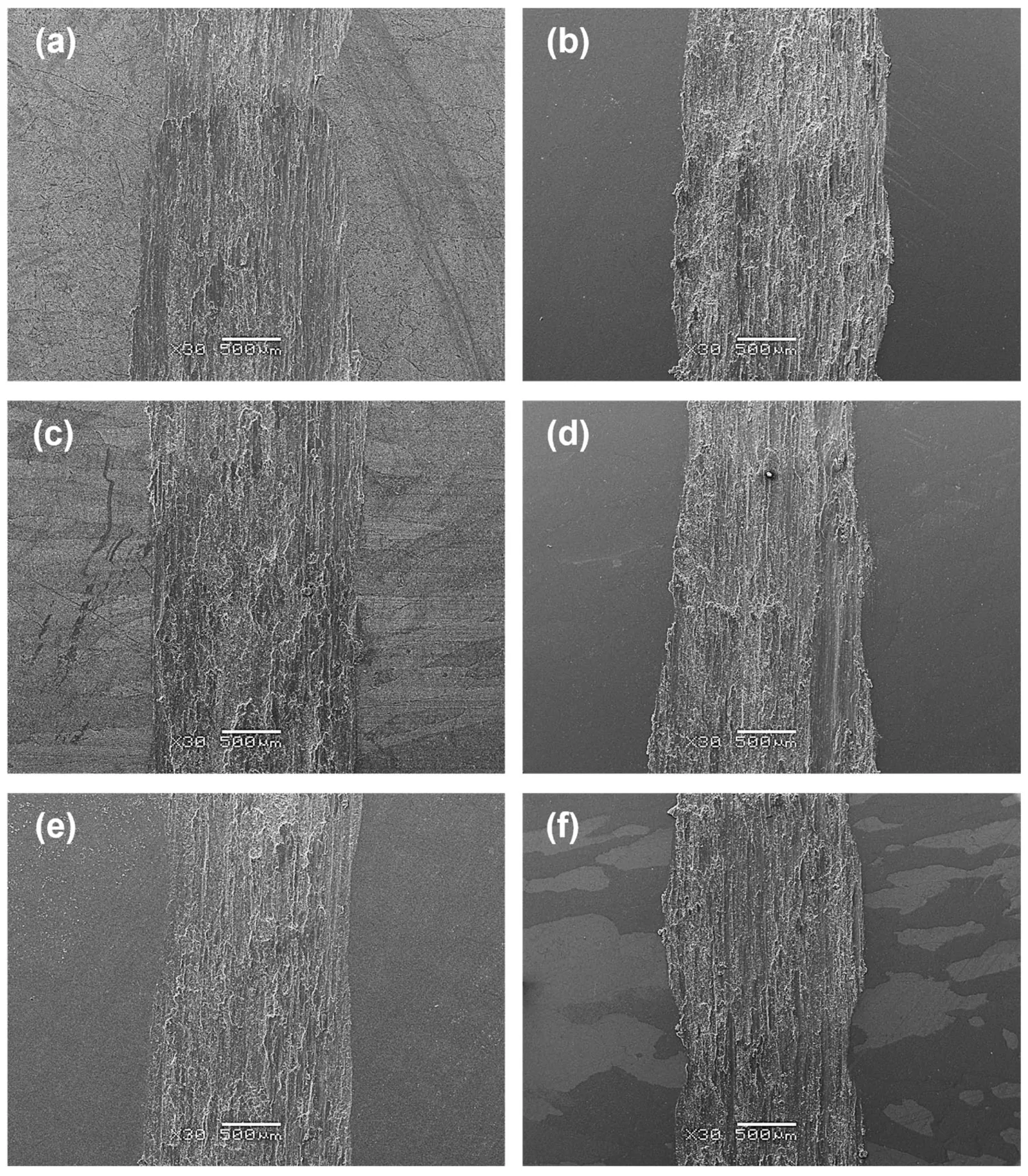
SEM images (×30) of wear tracks of samples: (**a**) Zr-10Nb, (**b**) (WQ)Zr-10Nb, (**c**) Zr-10Nb-2Mo, (**d**) (WQ)Zr-10Nb-2Mo, (**e**) Zr-10Nb-4Mo, (**f**) (WQ)Zr-10Nb-4Mo.

**Figure 7:**
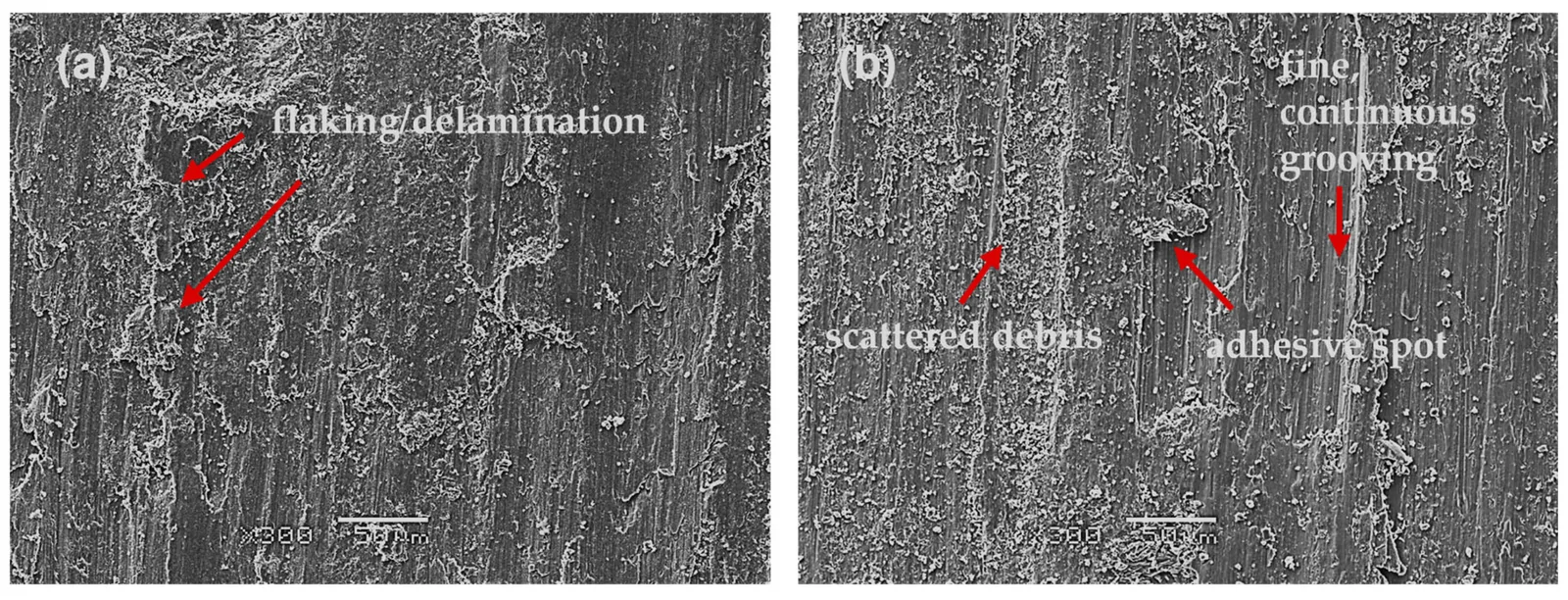
SEM images (×300) of wear tracks of samples: (**a**) Zr-10Nb-4Mo and (**b**) (WQ)Zr-10Nb-4Mo.

The tribological behavior of the investigated alloys under dry sliding conditions was evaluated in order to assess the influence of alloy composition and heat treatment on wear resistance. The obtained results enable a direct comparison of wear performance as a function of molybdenum content and water quenching. Mean values of the coefficient of friction (µ), cross-sectional area of wear tracks (A_w_), specific wear rates (w_s_), and the corresponding wear of the ZrO_2_ counter-samples are summarized in Table 5 and illustrated in Figure 4 and Figure 5. SEM observations of wear tracks are presented in Figure 6 and Figure 7.

Among the tested materials, the as-annealed Zr-10Nb alloy exhibited the highest mean wear, with a specific wear rate of w_s_ = 10.5 × 10^−4^ mm^3^/(N·m) and a mean wear-track cross-sectional area of A_w_ = 104,663 µm^2^. This composition was therefore used as the reference for evaluating the effects of molybdenum addition and water quenching. It was also the softest material among those tested (261 ± 35), although it showed the lowest mean coefficient of friction (µ = 0.588). Its higher wear coincided with the relatively coarse and heterogeneous microstructure observed in Figure 2a and Figure 3a, suggesting a possible relationship between microstructural refinement, hardness and wear resistance.

The addition of molybdenum was accompanied by a reduction in the mean wear parameters that became clear at the higher Mo content. Relative to Zr-10Nb, the mean specific wear rate decreased by approximately 5% for Zr-10Nb-2Mo (a difference within the experimental scatter) and by approximately 21% for Zr-10Nb-4Mo. A corresponding trend was observed for the wear-track cross-sectional area. This reduction coincided with the progressive microstructural refinement observed in Figure 2 and Figure 3 and with an increase in hardness, from 261 HV_IT_ for Zr-10Nb through 287 HV_IT_ to 330 HV_IT_ for Zr-10Nb-4Mo. The highest mean hardness among the initial specimens was recorded for Zr-10Nb-4Mo. However, the present measurements were performed on one specimen per material condition, and therefore the observed differences should be interpreted as differences between the tested specimens rather than as evidence of statistically established differences between independently produced material batches. In the as-annealed state, the coefficient of friction was practically unaffected by the Mo content (μ = 0.588, 0.607 and 0.595 for Zr-10Nb, Zr-10Nb-2Mo and Zr-10Nb-4Mo, respectively), so the lower wear of the 4Mo alloy was not accompanied by a clear change in friction. Such a correlation is consistent with previous reports describing a beneficial effect of grain refinement and increased hardness on wear resistance [23,24]. However, the present results demonstrate a correlation and do not by themselves establish a direct strengthening mechanism.

Lower mean wear values were observed after water quenching for all investigated compositions, although the magnitude of the change depended strongly on alloy composition. The mean specific wear rate decreased by approximately 6% for Zr-10Nb and Zr-10Nb-2Mo, whereas a substantially larger reduction of approximately 23% was observed for Zr-10Nb-4Mo. Water quenching was accompanied by a higher mean hardness for all compositions (Table 4). Consequently, (WQ)Zr-10Nb-4Mo exhibited the lowest mean specific wear rate, w_s_ = 6.4 × 10^−4^ mm^3^/(N·m), and the smallest wear-track cross-sectional area among the investigated samples, together with the highest mean hardness (354 ± 26). This behavior coincided with the refined and more homogeneous microstructure observed after quenching. At the same time, the relationship between hardness and wear was not strictly monotonic: (WQ)Zr-10Nb-2Mo was harder than as-annealed Zr-10Nb-4Mo, yet showed a higher specific wear rate. Hardness therefore does not explain the wear behavior alone, and other factors, such as microstructural homogeneity and the stability of the sliding contact, are likely to contribute. Water quenching increased the mean coefficient of friction for all compositions (from 0.588 to 0.694 for Zr-10Nb, from 0.607 to 0.678 for Zr-10Nb-2Mo, and from 0.595 to 0.633 for Zr-10Nb-4Mo), i.e., in spite of the lower wear. Consequently, (WQ)Zr-10Nb-4Mo, which showed the lowest wear, did not show the lowest friction (μ = 0.633), while the highest coefficient of friction (μ = 0.694) was recorded for (WQ)Zr-10Nb, which did not show the highest wear. In the investigated system, the specific wear rate therefore did not follow the coefficient of friction (Table 5). Low-magnification SEM observations (Figure 6) show wear tracks oriented along the sliding direction. Higher-magnification observations of Zr-10Nb-4Mo and (WQ)Zr-10Nb-4Mo (Figure 7) reveal longitudinal grooves together with localized flaking, scattered debris, and areas of material build-up. The longitudinal grooves are consistent with an abrasive contribution to the wear process. Local flaking, debris, and material accumulation indicate that additional wear processes may also contribute to material removal. However, SEM observations alone do not allow the individual wear mechanisms to be unambiguously distinguished. The quenched Zr-10Nb-4Mo sample exhibits a more uniform wear-track morphology and less severe localized surface damage, consistent with its lower mean specific wear rate (Table 5). The specific wear rate of the ZrO_2_ counter-samples ranged from 0.09 to 0.25 × 10^−4^ mm^3^/(N·m). For the Zr-10Nb-4Mo pair, water quenching was accompanied by a decrease in the counter-body wear rate from 0.25 to 0.20 × 10^−4^ mm3/(N·m), together with a decrease in the wear rate of the metallic specimen from 8.3 to 6.4 × 10^−4^ mm^3^/(N·m). These counter-body results should be interpreted descriptively, since the present data do not provide sufficient evidence to determine changes in the contribution of adhesive wear or in sliding-contact stability [25].

## 4. Discussion and Conclusions

XRD analysis revealed diffraction patterns consistent with the presence of bcc and hcp Zr-Nb-based solid solutions in all investigated alloys. No additional crystalline phases were identified within the detection limits of the applied method. Rietveld refinement using the two-phase structural model indicated an increase in the relative fraction of the hcp phase following water quenching. However, the precise chemical compositions of the identified phases and the possible presence of minor Mo-rich constituents could not be established from the present XRD data alone [26].

The absence of separately identifiable Mo-containing phases does not exclude the incorporation of molybdenum into the existing solid solutions [27,28]. The lattice parameter of the bcc phase decreased systematically with increasing nominal Mo content in both the initial and quenched specimens. This trend is compatible with changes in substitutional alloying and phase chemistry; however, the actual distribution of Mo between the phases was not measured. Changes in lattice parameters may also reflect residual strain and other microstructural effects. Therefore, the observed lattice contraction should not be treated as direct proof of a specific substitution mechanism. Nevertheless, increasing molybdenum content affects lattice distortion and the size of diffracting domains, which is reflected in peak broadening observed in the diffraction patterns [29]. Water quenching had a much stronger effect on the phase composition than Mo addition: the fraction of the hexagonal phase increased from about 50 to about 60 wt.%, and the lattice parameters of both phases increased (Table 2 and Table 3). This may result from the rapid cooling, which retains a state far from equilibrium, and from quenching-induced strain. The contributions of strain and chemical composition were not separated in the present study. These results suggest that molybdenum is accommodated within the existing solid solution, influencing the crystal lattice without altering the qualitative phase composition.

Although no additional crystalline phases were identified, molybdenum addition and water quenching were associated with visible changes in microstructural morphology. The OM and SEM images suggest a tendency toward finer microstructural features, particularly in some of the Mo-containing quenched specimens. Such changes may be associated with the influence of alloying elements on phase transformation kinetics and grain-boundary mobility. However, the grain size and chemical distribution were not quantified, and the mechanisms responsible for the observed morphology remain to be established.

In addition to changes in microstructural morphology, contrast variations were observed near some microstructural boundaries in the OM and SEM images (Figure 2 and Figure 3). Microstructural evolution during thermal processing of zirconium-based alloys has also been discussed in the literature [21,30]. The observed contrast variations may reflect local differences in phase constitution, crystallographic orientation, or chemical composition; however, these contributions were not independently identified. Following water quenching, the micrographs suggested a more uniform distribution of microstructural features, although boundary-associated contrast remained visible in some regions.

The mean specific wear rates tended to decrease with increasing molybdenum content and following water quenching, with the lowest value recorded for the (WQ)Zr-10Nb-4Mo alloy. The coefficient of friction varied within a relatively narrow range (μ = 0.588–0.694, Table 5) and did not follow the same trend as the mean wear rate. In particular, water quenching was associated with an increase in the mean coefficient of friction despite a decrease in the mean specific wear rate. This suggests that the observed differences in wear behavior cannot be explained solely by changes in friction. SEM observations of the wear tracks revealed longitudinal grooves oriented along the sliding direction, indicating a possible abrasive contribution to wear. Local flaking, wear debris, and material accumulation suggest that additional material-removal mechanisms may also be involved. Oxidative wear may contribute to the process, particularly considering the tendency of zirconium-based alloys to form surface oxides during sliding in air against ZrO_2_; however, this mechanism was not experimentally verified in the present study. The relatively uniform worn-surface morphology of the (WQ)Zr-10Nb-4Mo alloy coincided with its lowest mean specific wear rate. The investigated specimens exhibited a general association between higher mean indentation hardness, changes in microstructural morphology, and lower mean specific wear rates. In particular, the (WQ)Zr-10Nb-4Mo alloy exhibited the highest mean instrumented hardness (354 HV_IT_) and the lowest mean specific wear rate (6.4 × 10^−4^ mm^3^/(N·m)). Previous studies have also demonstrated that mechanical properties and microstructural characteristics can influence the wear behavior of zirconium-based and other metallic alloys [23,31,32,33]. Nevertheless, the relationship between mean hardness and specific wear rate was not strictly monotonic across all investigated conditions, and the available results do not establish a direct causal relationship. Furthermore, only one specimen was subjected to tribological testing for each material condition, while the reported variability was determined from repeated measurements along individual wear tracks. Consequently, the observed differences should be considered preliminary and require confirmation through independently replicated experiments. Further quantitative microstructural characterization and complementary investigations of wear mechanisms would also be necessary to establish the mechanisms governing the observed tribological behavior.

ZrO_2_ was selected as a hard ceramic counter-body relevant to comparative tribological testing of biomedical metallic materials [34,35]. However, the present experiments were conducted under dry sliding conditions and do not reproduce a specific in vivo tribological pair or the physiological environment. The results should therefore be interpreted primarily as a comparative assessment of the wear behavior of the investigated alloys. Under lubricated conditions involving simulated body fluids or serum-containing solutions, friction and wear may differ substantially from those measured under dry sliding conditions because of lubrication, protein adsorption and possible tribochemical interactions. The magnitude and direction of these changes cannot be predicted from the present experiments and require dedicated testing under physiologically relevant conditions. This will be examined in future work.

In summary, molybdenum addition and water quenching were associated with microstructural refinement and a reduction in mean dry-sliding wear, with the lowest wear observed for the water-quenched Zr-10Nb-4Mo alloy. SEM observations indicate an abrasive contribution to wear together with additional local material-removal processes. The results provide a basis for further evaluation of these alloys under physiologically relevant tribological conditions, together with complementary mechanical, corrosion, and biological characterization.

## Figures and Tables

**Table 1:** Compositions of the studied samples.

Sample Identification	Element Content [wt.%]		
	Zr	Nb	Mo
Zr-10Nb	90	10	0
(WQ)Zr-10Nb			
Zr-10Nb-2Mo	88	10	2
(WQ)Zr-10Nb-2Mo			
Zr-10Nb-4Mo	86	10	4
(WQ) Zr-10Nb-4Mo			

**Table 2:** Quantitative phase analysis of studied samples using the Rietveld method—lattice parameters [nm].

Phase		ICDD	Initial Samples (Mo wt.%)			Water-Quenched Samples (Mo wt.%)		
			0%	2%	4%	0%	2%	4%
**bcc Zr-Nb**	a_0_	0.3524	0.3536 (1)	0.3530 (1)	0.3511 (1)	0.3553 (1)	0.3538 (1)	0.3526 (1)
**hcp Zr-Nb**	a_0_	0.3216	0.3246 (1)	0.3247 (1)	0.3248 (1)	0.3252 (1)	0.3255 (1)	0.3254 (1)
	c_0_	0.5134	0.5175 (1)	0.5177 (1)	0.5178 (1)	0.5189 (1)	0.5189 (1)	0.5191 (1)

**Table 3:** Phase fractions (wt.%) estimated by Rietveld refinement using the two-phase bcc/hcp structural model.

Phase	Initial Samples (Mo wt.%)			Water-Quenched Samples (Mo wt.%)		
	0%	2%	4%	0%	2%	4%
**bcc Zr-Nb**	49.5 (2)	50.0 (2)	50.2 (2)	37.5 (2)	38.6 (2)	39.7 (2)
**hcp Zr-Nb**	50.5 (2)	50.0 (2)	49.8 (2)	62.5 (2)	61.4 (2)	60.3 (2)

**Table 4:** Instrumented Vickers hardness (HV_IT_) of the investigated alloys (mean ± standard deviation, *n* = 10 indentations per specimen).

Sample Identification	HV_IT_
**Zr-10Nb**	261 ± 35
**(WQ)Zr-10Nb**	296 ± 52
**Zr-10Nb-2Mo**	287 ± 10
**(WQ)Zr-10Nb-2Mo**	346 ± 52
**Zr-10Nb-4Mo**	330 ± 25
**(WQ)Zr-10Nb-4Mo**	354 ± 26

**Table 5:** Average tribological parameters of the tested samples under dry sliding conditions.

Sample Identification	Analyzed Parameter			
	*Coefficient of Friction* (µ)	*Cross-Sectional Area of Wear Tracks* (A_w_) [µm^2^]	*Specific Wear Rate* (w_s_) (×10^−4^) [mm^3^/N∙m]	*Specific Wear Rate of ZrO*_2_*Counter-Samples* (×10^−4^) [mm^3^/N∙m]
**Zr-10Nb**	0.588	104,663 ± 7520	10.5 ± 0.8	0.09
**(WQ)Zr-10Nb**	0.694	98,775 ± 10,521	9.9 ± 1.1	0.11
**Zr-10Nb-2Mo**	0.607	99,538 ± 9586	10.0 ± 1.0	0.11
**(WQ)Zr-10Nb-2Mo**	0.678	93,638 ± 15,887	9.4 ± 1.6	0.11
**Zr-10Nb-4Mo**	0.595	83,138 ± 9588	8.3 ± 1.0	0.25
**(WQ)Zr-10Nb-4Mo**	0.633	64,084 ± 10,082	6.4 ± 1.0	0.20

## Data Availability

The original contributors presented in the study are included in the article, further inquiries can be directed to the corresponding author.
